# Potential of zebrafish as a model to characterise MicroRNA profiles in mechanically mediated joint degeneration

**DOI:** 10.1007/s00418-020-01918-1

**Published:** 2020-09-15

**Authors:** Elizabeth A. Lawrence, Chrissy L. Hammond, Emma J. Blain

**Affiliations:** 1 School of Physiology, Pharmacology and Neuroscience, University of Bristol, Bristol, BS8 1TD UK; 2 Biomechanics and Bioengineering Centre Versus Arthritis, School of Biosciences, Cardiff University, Cardiff, CF10 3AX UK

**Keywords:** Zebrafish, Joints, Loading, Cartilage, MicroRNA, Osteoarthritis

## Abstract

Mechanically mediated joint degeneration and cartilage dyshomeostasis is implicated in highly prevalent diseases such as osteoarthritis. Increasingly, MicroRNAs are being associated with maintaining the normal state of cartilage, making them an exciting and potentially key contributor to joint health and disease onset. Here, we present a summary of current in vitro and in vivo models which can be used to study the role of mechanical load and MicroRNAs in joint degeneration, including: non-invasive murine models of PTOA, surgical models which involve ligament transection, and unloading models based around immobilisation of joints or removal of load from the joint through suspension. We also discuss how zebrafish could be used to advance this field, namely through the availability of transgenic lines relevant to cartilage homeostasis and the ability to accurately map strain through the cartilage, enabling the response of downstream MicroRNA targets to be followed dynamically at a cellular level in areas of high and low strain.

## Introduction

Skeletal homeostasis is intrinsically linked to mechanical loading, with physiological loading promoting cartilage health (Lee and Bader 1997; Otterness et al. 1998; Soltz et al. 2000; Manninen 2001; Galois et al. 2003; Shelton, Bader and Lee 2003; Sharma, Saxena and Mishra 2007) and maintenance of bone mass (Russo 2009). Abnormal loading of joints, particularly the hip (Croft et al. 1992) and knee (Felson et al. 1991; Coggon et al. 2000), is associated with joint degeneration and osteoarthritis (OA) onset. OA is the most common joint disease globally, with up to 50% of people over the age of 65 estimated to suffer from the disease (Lawrence et al. 2008; Murphy et al. 2008). During OA, the articular cartilage of joints is degraded leading to ectopic bone formation, joint inflammation and severe pain in sufferers, with almost 75% of people living with OA experiencing constant pain and 12.5% describing their pain as frequently unbearable (Arthritis Research UK 2017).

**Fig. 1:**
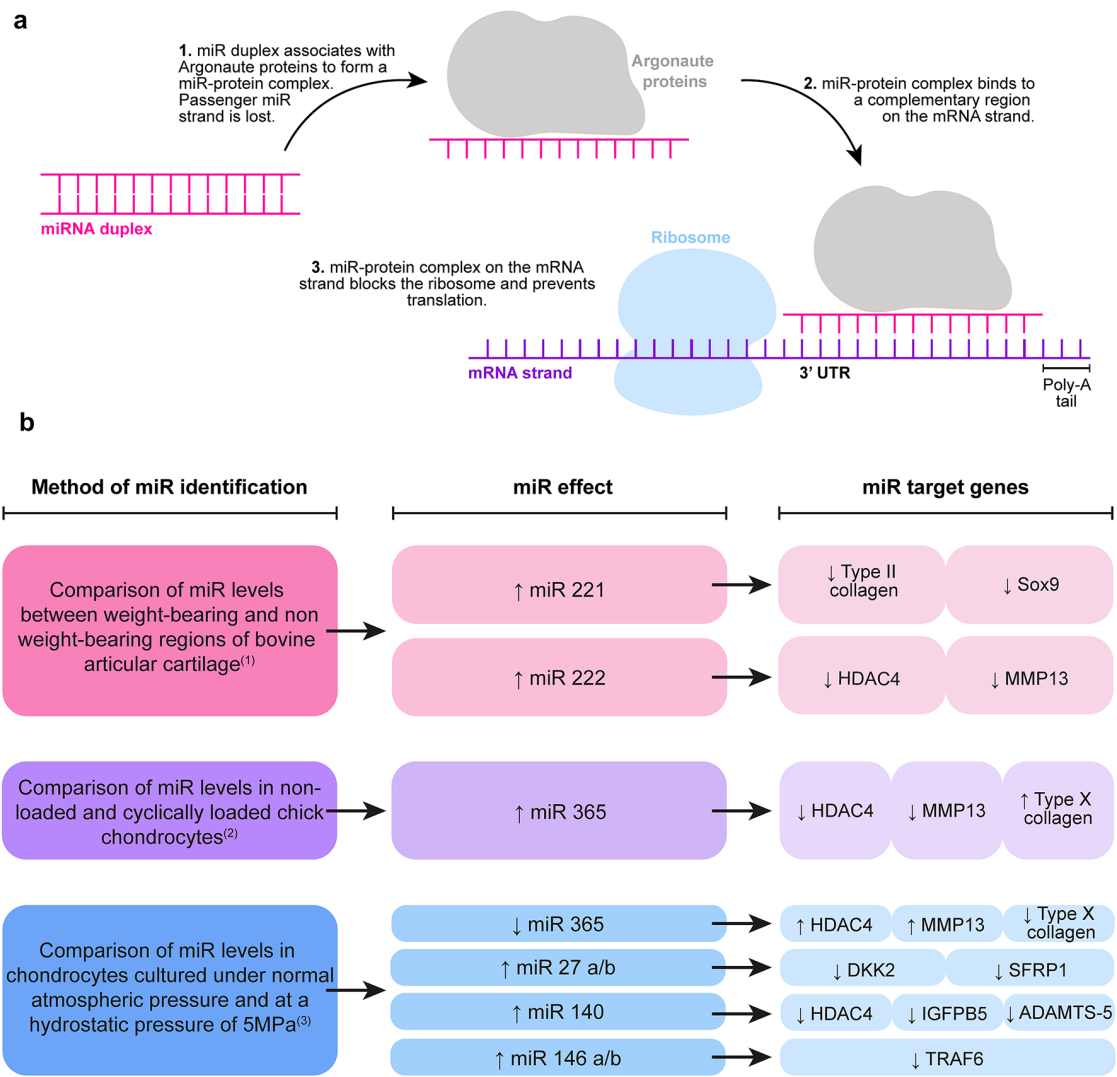
**a** Schematic overview of miR interaction with mRNA. **b** Summary of miRs known to respond to specific stimuli in chondrocytes, and the genes which are affected by changes to these miR expression levels. The mechanical stimulus found to alter miR expression is listed on the left, the miR and how its expression is altered is displayed in the central column and the downstream gene targets of each miR are shown in the right column. ^(1)^Dunn et al. 2009, ^(2)^Guan et al. 2011, ^(3)^Cheleschi et al. 2017

Although changes to mechanical loading have been identified as a major risk factor for OA (Kujala et al. 1995; Lane et al. 1999; McAlindon et al. 1999), the full mechanism by which mechanically mediated joint degeneration occurs during OA onset is not fully understood. It has been suggested that expression of short non-coding MicroRNAs (miRs) which can regulate gene expression and are key in cartilage homeostasis could be key to disease onset. miRs have an average length of 22 nucleotides (O’Brien et al. 2018) and are able to interact with mRNAs, mostly via the 3’ untranslated region (UTR), to modify their expression (Fig. 1a) (Ha and Kim 2014). Alterations in articular cartilage miR expression are associated with joint degeneration and OA pathogenesis (Araldi and Schipani 2010; Goldring and Marcu 2012; Swingler et al. 2012); importantly, miRs are reported to be mechanically regulated in cartilage chondrocytes.

## Mechano-regulation of miRs and their downstream targets in chondrocytes

Early evidence of mechanically induced epigenetic regulation in articular cartilage was reported in stifle joint medial femoral condyles with increased expression of miR-221 and miR-222 in anterior weight-bearing compared to posterior non-weight-bearing regions (Dunn et al. 2009). Putative gene targets for miR-221 and miR-222 (contains conserved seed site) include histone deacetylase 4 (HDAC4) and matrix metalloproteinase 13 (MMP13) (Song et al. 2015). miR-221 expression inversely correlates with expression of chondrogenic markers including type II collagen and the transcription factor SOX9 in mesenchymal stem cells (Lolli et al. 2014)); thus, it may have relevance in regulating load-induced cartilage homeostasis.

Significant elevation of miR-365 has previously been observed after application of tensile strain to 3D-sponge scaffolds seeded with chick chondrocytes (Guan et al. 2011) and human chondrocytes isolated from macroscopically normal regions of OA cartilage (Yang et al. 2016). A direct target gene is HDAC4 which regulates downstream molecules including MMP13 and type X collagen (Yang et al. 2016).

Significant induction of miR-27a/b, miR-140 and miR-146a/b with a concomitant reduction in miR-365 was observed in OA chondrocytes exposed to hydrostatic pressure (Cheleschi et al. 2017). Other studies have corroborated the mechano-regulation of miR-146a in chondrocytes (Jin et al. 2014; Guan et al. 2018) and miR-27 (Blain, unpublished observations). WNT signalling molecules *e.g.* dickkopf-2 (DKK2) and secreted frizzled-related protein 1 (sFRP1) have been identified as miR-27 target genes (Wu et al. 2019), and more recently, the TNF receptor-associated factor-6 (TRAF-6)-mediated NFκB signalling pathway has been shown to be targeted by miR-146 (Shao et al. 2020). miR-140, a key regulator of chondrogenesis and cartilage homeostasis (Miyaki et al. 2010), has many putative target genes, but of relevance to this perspective is its known modulation of HDAC4, insulin growth factor-binding protein 5 (IGFBP5) and a disintegrin and metalloproteinase with thrombospondin motif 5 (ADAMTS-5) (Tuddenham et al. 2006; Cheleschi et al. 2017), all of which are molecules important in maintaining cartilage homeostasis. A summary of mechano-regulated miRs and their downstream targets in chondrocytes can be found in Fig. 1b.

## Existing model systems for investigating involvement of mechanical load in joint degeneration

Many models have been developed, involving both in vitro and in vivo systems, to investigate the role of abnormal/altered mechanical load in mediating degeneration of the synovial joint tissues that results in initiation and progression of OA.

### In vitro loading models

Development of in vitro systems for mechanobiology research has utilised isolated cells derived from several species typically including human, mouse, bovine and porcine as either primary or cell lines; mechanical load is subsequently applied to these cells following culture as either a monolayer (Millward-Sadler et al. 2000; Ikenoue et al. 2003) or in 3D constructs (Buschmann et al. 1995; Roberts et al. 2001), or as a co-culture system with at least two different joint cell populations (McCorry, Puetzer and Bonassar 2016). Explant tissues, using only articular cartilage (Guilak et al. 1994) or alternatively an osteochondral plug (cartilage–bone unit) have also been utilised to investigate mechanobiological pathways (Blain et al. 2001; Patwari et al. 2003). There are distinct advantages to using in vitro culture systems to characterise cell behaviour in response to mechanical stimuli. Key amongst these is the ability to accrue a large cell population for experimentation, as well as co-culturing cells of different origins to investigate communication and interplay in response to loading. Use of explant tissue provides an in-situ environment which partially recapitulates an in vivo system due to the presence of an extensive, native extracellular matrix (ECM), facilitating cell–matrix communication, known to be critical in mechano-signalling (Guilak et al*.* 2006).

Utilisation of these in vitro models has contributed significantly to our understanding of how cells respond to mechanical stimuli and have facilitated characterisation of the molecular pathways that initiate tissue degeneration; however, what they cannot do is recapitulate the complex interactions that exist within the joint tissues, *i.e.* articular cartilage, bone, meniscus, synovium and associated vasculature. Furthermore, the interplay of mechanical responses with other biological stimuli, *e.g.* inflammation, or the longitudinal effects of mechanical stimuli on cell/tissue behaviour can only be comprehensively analysed in an in vivo model system.

### In vivo loading models

In vivo loading models have been developed to replicate the pathological features resulting from a joint injury or mechanical insult, often referred to as a model of post-traumatic OA (secondary OA). Such mechanical insult can either be administered surgically, *e.g.* via damage of the ligaments or meniscus, or via non-invasive means, *e.g.* application of external loads to the joint.

#### Surgical loading models

One of the earliest models that demonstrated the involvement of mechanical injury in induction of joint degeneration and OA pathology was the *Pond–Nuki* model (Pond and Nuki 1973); transection of the canine anterior cruciate ligament (ACL) altered the stability and biomechanics of the joint resulting in progression of OA. Subsequent studies involving ACL transection in other species including rabbits (Batiste et al. 2004), cats (Herzog et al. 1993) and sheep (Beveridge et al. 2013) have also been performed. Since then, ACL transection in rodent models has been reported with induction of tissue degeneration in response to altered joint biomechanics (Kamekura et al. 2005; Okamura et al. 2010).

Another common surgical approach to studying how joint tissue components respond to altered loading patterns is via meniscal destabilisation involving either partial or complete removal of the medial meniscus, with early studies performed in rabbits (Shapiro and Glimcher 1980) and guinea pigs (Meacock, Bodmer and Billingham 1990), and subsequently in mice (Glasson, Blanchet and Morris 2007). Although these models induce tissue degeneration due to altered joint biomechanics, the procedures themselves are invasive and require opening of the joint capsule; this in itself can initiate an inflammatory response impacting the local environment and may not be completely representative of the mechanical insult alone, thus necessitating increased animal numbers to provide “sham” controls (incision only surgeries).

#### Non-invasive loading models

In recent years, there has been an emergence in non-invasive mouse models of post-traumatic OA (PTOA). Such models are believed to more accurately recapitulate the mechanisms involved in mechanically induced injuries in humans, initiating joint degeneration through direct damage to the tissue components of the joint. These non-invasive models rely on the external application of a mechanical load to the tibia, either as a single traumatic insult or a defined period of loading, without any surgical intervention.

Utilisation of a single compressive load (12 N) has been previously demonstrated to rupture the ACL initiating pathological changes in murine articular cartilage and underlying subchondral bone (Christiansen et al. 2012). Pathologically distinct phases with an early inflammatory phase and a later degenerative component are evident in the non-invasive murine PTOA model (Gilbert et al. 2018); this proves advantageous as a model system as it provides a ‘therapeutic window’ following mechanical insult in which to assess treatments to delay or halt joint degeneration.

Tibial compression, via the application of cyclic axial compressive load transmitted through the natural articulation of the murine knee joint, induces articular cartilage overloading and tissue damage, following multiple loading episodes (Poulet et al. 2011). These non-invasive loading models have also been used to investigate the interplay of mechanical load and genetics, with use of genetically engineered mice (gene knockouts and transgenic overexpression) to more clearly delineate the importance of these risk factors in joint degeneration. Overall, the non-invasive loading models obviate the need for technically challenging surgical techniques, but more importantly avoid any confounding effects induced by the trauma of the surgical procedure and are more translatable to mechanical trauma experienced by humans.

### Models of unloading

The absence of weight-bearing is equally detrimental to the health of the joint tissues demonstrating that a physiological range of mechanical load is essential for maintenance of homeostasis. Numerous in vivo models of joint ‘unloading’ or immobilisation have been utilised to characterise how the absence of loading affects tissue behaviour, particularly in articular cartilage. Seminal early studies induced knee joint immobilisation using casts or surgery (limb amputation), typically in canine models (Palmoski, Perricone and Brandt 1979; Palmoski, Colyer and Brandt 1980), rabbits (Sood 1971; Langenskiöld, Michelsson and Videman 1979) and later in rodents (Hagiwara et al. 2009). An alternative model involving suspension of rodents by the tail has also been used to recapitulate hindlimb unloading (Tomiya et al. 2009; Nomura et al. 2017). Joint unloading inhibited expression of key ECM components, *e.g.* proteoglycans, reduced cartilage thickness and induced surface fibrillations, resulting in disruption of articular cartilage integrity. Interestingly, effects were reversible following joint ‘reuse’ and the reintroduction of weight-bearing (Palmoski, Perricone and Brandt 1979; Behrens, Kraft and Oegema 1989), demonstrating the highly adaptive nature of articular cartilage.

## Prospects for using zebrafish as a model for mechanically mediated joint degeneration to investigate miR dysregulation

Increasingly, the freshwater teleost zebrafish is used as a laboratory model for disease with the low cost of housing, ease of genetic manipulation, rapid development (which has been carefully staged (Kimmel et al. 1995)) and genetic tractability among the major advantages of this model. Although zebrafish are more remote from humans than other animal models (last common ancestor was 445 million years ago compared to 96 million years ago for rodents (Ali et al. 2011)) and the fact they have undergone genome duplication (Meyer and Schartl 1999): many genes are conserved (Dodd et al. 2000; Taylor et al. 2003) with around 70% of human genes found to have at least one orthologue (Howe et al. 2013) and 85% of disease-related genes being conserved (Wellcome Trust 2013) in zebrafish. Generally, zebrafish are ideal model organisms for studying disease as they have transparent larvae which enable dynamic longitudinal in vivo imaging in the same fish throughout maturation. They have high fecundity with the eggs developing externally, meaning the mother does not have to be killed to study early development, thus reducing animal costs and complying with the 3Rs principles of reduction and replacement (Russell and Burch 1959). In addition to these more general advantages, a number of specific factors are discussed below which make zebrafish an attractive prospect for studies into the role of miRs in mechanically mediated joint degeneration.

### Zebrafish cartilage contains components also found in human articular cartilage. Loading of cartilage can be manipulated in zebrafish

**Fig. 2:**
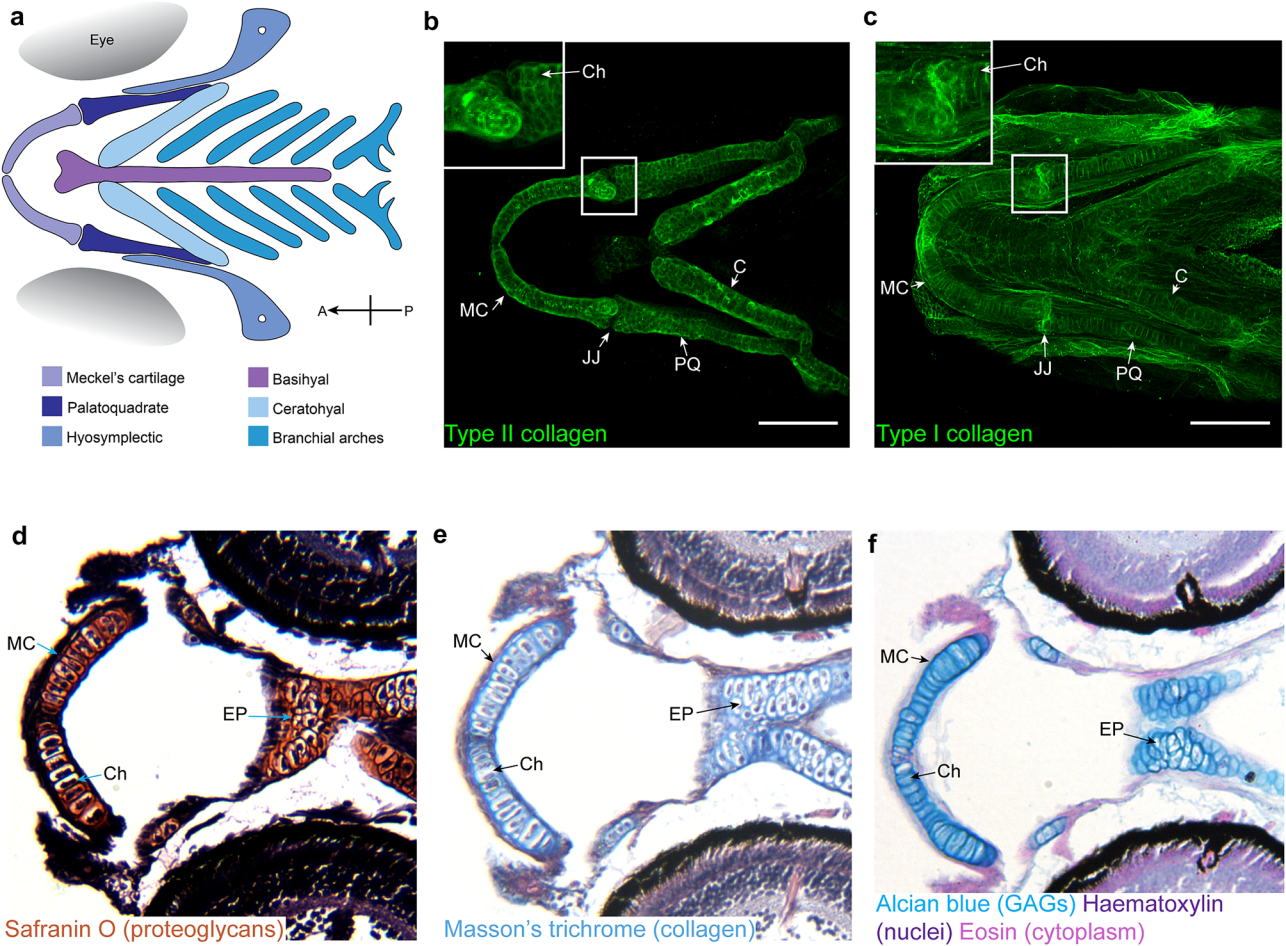
Zebrafish craniofacial cartilage has key components found in human articular cartilage. **a** Schematic representation of the cartilage elements which make up the zebrafish craniofacial skeleton at 5 dpf. Orientation compass included in bottom right, with *A* anterior and *P* posterior. **b**, **c** Ventral view confocal image stacks of 5 dpf craniofacial skeleton immunostained for type II collagen (**b**) and type I collagen (**c**) in the ECM surrounding chondrocytes. Inset shows zoom of jaw joint. Scale bar = 100 μm. Images in b and c previously published in (Lawrence et al. 2018). **d–f** Ventral paraffin sections of 5 dpf craniofacial skeleton stained with Safranin O (**d**) to show proteoglycans, Masson’s trichrome (**e**) to show the presence of collagen, and alcian blue (**f**) to show the presence of glycosaminoglycans in the chondrocyte ECM which makes up the cartilage. *MC* Meckel’s cartilage, *JJ* jaw joint, *PQ* palatoquadrate, *C* ceratohyal, *Ch* example of a chondrocyte surrounded by ECM, *EP* ethmoid plate

At 5 days post fertilisation (dpf) the zebrafish craniofacial skeleton is made up of distinct cartilaginous elements (Fig. 2a) derived from the migratory neural crest (Schilling and Kimmel 1994). This cartilage contains all the key components of human cartilage including chondrocytes which undergo hypertrophy (Mitchell et al. 2013; Askary et al. 2016), collagens and proteoglycans (Fig. 2b–f).

**Fig. 3:**
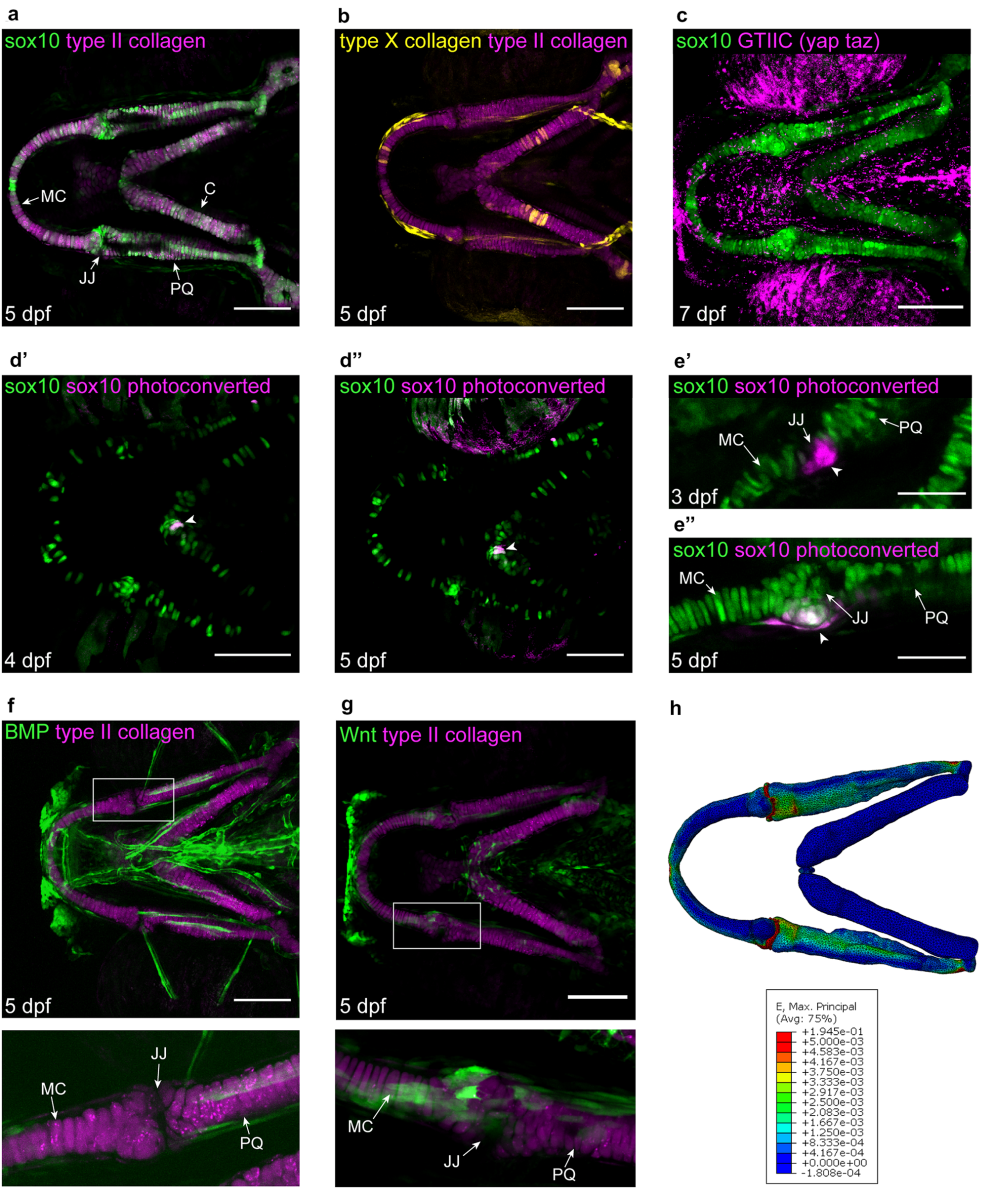
Tools available in zebrafish to facilitate spatiotemporal study of miRNA targets at a cellular level. **a–c** Ventral view confocal images of the craniofacial cartilages of *Tg(4.9Sox10:EGFP;col2a1aBAC:mcherry)* (**a**), *Tg(col2a1aBAC:mcherry;col10a1aBAC:citrine)hu7050* (**b**), *Tg(- 4.9Sox10:EGFP; 4xGTIIC:eGFP)* (**c**) imaged live. Scale bar = 100 μm. **d’**, **d’’** Ventral view confocal images of the craniofacial cartilages of *Tg(Sox10:GAL4- VP16; UAS:Kaede)* with photoconverted chondrocytes shown in magenta and annotated with a white arrowhead in the same fish at 4 dpf (**d’**) and 5dpf (**d’’**). Scale bar = 100 μm. **e’–e’’** Confocal images of the lower jaw joint in *Tg(Sox10:GAL4- VP16; UAS:Kaede)* with photoconverted chondrocytes shown in magenta and annotated with a white arrowhead in the same fish at 3 dpf (**e’**) and 5dpf (**e’’**). Scale bar = 50 μm, images in e’ and e’’ previously published in (Brunt et al. 2017). **f**, **g** Ventral view confocal images of the craniofacial cartilages of *Tg(5xBMPRE-Xla.Id3:GFP; col2a1aBAC:mCherry)* (**f**) and *Tg(7xTCF.XlaSiam:nlsGFP;col2a1aBAC:mCherry)* (**g**) imaged live. Scale bar = 100 μm. **h** Finite element model of maximum principal (E. max) strains in 5dpf wild-type zebrafish viewed from a dorsal orientation. E. max represents tensional strains with cooler colours (blue) on the scale corresponding to lower strain and red corresponding to higher strain values. *MC* Meckel’s cartilage, *JJ* jaw joint, *PQ* palatoquadrate, *C* ceratohyal

As with human cartilage, the craniofacial cartilages of larval zebrafish have been shown to be mechanically sensitive (Brunt et al. 2016). The biomechanical load exerted on the craniofacial cartilages and joint can be controlled in larvae genetically or pharmacologically to induce paralysis (either flaccid or rigid) to reduce load, or the induction of hyperactivity to induce excessive loading conditions (Shwartz et al. 2012; Brunt et al. 2016). Strains in the zebrafish skeleton, as in other systems, can be modelled computationally using Finite Element Analysis (FEA) (Fig. 3h), a computational technique that predicts deformation, stress and strain in a structure when subjected to external loading conditions (Bright and Rayfield 2011). FEA can be used to visualise patterns of strain by displaying them with colours to show areas of high and low strain, or degree of tissue deformation. Finite element models for the developing zebrafish jaw have been published for wild-type fish (Brunt et al. 2016; Brunt et al. 2016), mutants (Lawrence et al. 2018) and larvae exposed to different gravitational fields (Lawrence et al. 2020). Cartilage material properties change during development, in response to mutations to chondrocyte genes or changes to gravitational force, and can be tested ex vivo by atomic force microscopy (AFM) or nanoindentation (Lawrence et al. 2018, 2020), to provide further information and accuracy to FE models.

Cavitation of the jaw joint between the Meckel’s cartilage and the palatoquadrate to form a fluid-filled synovium (as opposed to a cellular interzone) occurs late in zebrafish (around 14 dpf) relative to onset of joint movement; this joint continues to mature, eventually forming a synovial joint with all of the tissues observed in a human synovial joint (Askary et al. 2016). As the cartilaginous craniofacial skeleton forms early in development and many elements are retained into adulthood, it is a valuable model to investigate at a cellular level how the cartilage is affected by different genetic mutations, such as those to downstream miR targets, and environmental factors, such as mechanical loading.

The presence of lower jaw cartilages in which loading can be manipulated allows miRs which are responsive to mechanical load to be isolated (Chen et al. 2005) and identified so that specific pathways can be dysregulated through CRISPR/cas9-induced mutation of miR signalling pathway components or through immersion in miR agonists/antagonists and the effect on chondrocytes studied. Other skeletal elements in zebrafish, such as the vertebral centra, have also been demonstrated to be mechanically sensitive, and to undergo remodelling in response to load (Fiaz et al. 2012; Ofer et al. 2019), which could be regulated by miRs. Finite element models also exist for the zebrafish vertebral column (Newham et al. 2019; Ofer et al. 2019). However, as patterning and mineralisation of the vertebral column occur somewhat later than that of the jaw (from 8dpf onwards) (Wopat et al. 2018), testing the role of specific miRs in the vertebral column is likely to be more technically challenging.

### Tools available in zebrafish which allow for cellular changes to be observed in the context of the whole tissue

A major advantage to zebrafish as a model is the possibility to dynamically image cellular events which could be impacted by miR dysregulation such as chondrocyte behaviour, ECM production and the expression of targets downstream of the mechanically regulated miR in the living zebrafish. This can be achieved through high-resolution confocal or lightsheet imaging of transgenic reporter lines.

Many transgenic lines (listed in full in Table 1) relevant to the study of cartilage homeostasis and degeneration exist, including lines which facilitate the study of chondrocyte behaviour and expression of important ECM components such as the *sox9:eGFP*, *sox10:eGFP*, *col2:mCherry* and *colx:citrine* lines. As discussed, these molecules have been implicated as downstream miR targets. In the context of zebrafish, they enable chondrocyte morphology and migration within the cartilage to be tracked throughout maturation from immature stages when *sox9* and *sox10* are predominantly expressed (Fig. 3a), to hypertrophic stages just prior to ossification when chondrocytes express *colX* (Schmid et al. 1991; Mitchell et al. 2013);(Fig. 3b). In later larval stages, the impact of load on osteogenesis can be studied dynamically, for example with the *GTIIC:eGFP* line which shows Yap/Taz – TEAD activity (Fig. 3c). Photoconvertible transgenics such as *sox10:kaede* offer the opportunity to track single cells (Fig. 3d’–e’’) to study how their migration and maturation are affected depending on their location in the cartilage and the forces they are exposed to.

**Table 1:** Full nomenclature of transgenic lines important for study of mechanically mediated joint degeneration

Text reference	Full transgenic name	References
*sox9:eGFP*	*Tg*(*− 2421/* + *29sox9b:EGFP*_uw2_)	Garcia et al. (Garcia et al. 2017)
*sox10:eGFP*	*Tg(- 4.9Sox10:EGFP)*	Wada et al. (Wada et al. 2005)
*col2:mCherry*	*Tg(col2a1aBAC:mcherry)*	Hammond and Schulte-Merker (Hammond and Schulte-Merker 2009)
*colx:citrine*	*Tg(col10a1aBAC:citrine)hu7050*	Mitchell et al. (Mitchell et al. 2013)
*GTIIC:eGFP*	*Tg(4xGTIIC:EGFP)*	Miesfield and Link 2014 (Miesfeld and Link 2014)
*sox10:kaede*	*Tg(Sox10:GAL4- VP16;UAS:Kaede)*	Hatta et al. (Hatta, Tsujii and Omura 2006)
*wnt:GFP*	*Tg(7xTCF.XlaSiam:nlsGFP)*	Moro et al. (Moro et al. 2012)
*BMP:GFP*	*Tg(5xBMPRE-Xla.Id3:GFP)*	Alexander et al. (Alexander et al. 2011)

Zebrafish represent a model in which the spatiotemporal response of both cells and genes to a mechanical stimuli can be measured simultaneously. Finite Element modelling can be used to measure the response of specific cell populations, with areas of altered strain having been mapped previously (Fig. 3h) and changes to chondrocyte morphology and ECM composition in these regions analysed (Lawrence et al. 2020). Live imaging of transgenic reporter lines such as the *wnt:GFP* or the *BMP:GFP* reporter (Fig. 3f, g) also enables the response of specific genes to be tracked in regions of abnormal strain. This technique was used to correlate regions of high strain in joint morphogenesis with *Wnt* reporter expression, leading to the identification of *Wnt16* as a mechanoresponsive gene that controls joint cell behaviour (Brunt et al. 2017). This allows the effects of a genetic mutation or pharmacological intervention to be mapped according to where in the cartilage a chondrocyte is, for example at the joint or at a muscle attachment site, to see if some populations of cells react differently to stimuli.

In addition to the transgenic lines available, there is also good availability of miR tools in zebrafish to enable study of joint degeneration in the context of mechanically mediated miRs. miRs have been shown to play a role in cartilage development and homeostasis in zebrafish. Zebrafish of the *dicer1* mutant line, which lacks the Dicer miR processing enzyme, have abnormal craniofacial development and increased expression of *sox10* (Weiner et al*.* 2019), confirming that miRs are essential for normal development (Wienholds et al. 2003). A number of miRs such as miR-140 and miR-29 are regulated by *sox9* in zebrafish, and in turn control aspects of chondrocyte behaviour (Nakamura et al. 2012; Le et al. 2016), while others such as miR-92a maintain BMP signalling in cartilage. The spatiotemporal expression of a number of miRs in zebrafish has also been determined using techniques such as in-situ hybridisation and microarrays (Wienholds et al. 2005). This includes the mapping of 115 miRs which are conserved from vertebrates (Wienholds et al. 2005).

Synthetic miRs have successfully been used to downregulate genes of interest in zebrafish (Giacomotto, Rinkwitz and Becker 2015), indicating that this model system is tolerant to miR injection to target downstream molecules. This would enable specific miR signalling pathways to be inhibited in existing transgenic lines and the resulting changes to cells in areas of known strain to be studied dynamically.

We conclude that despite the existence of several model systems to study the role of mechanical loading in joint degeneration, and the emergence of non-invasive mouse models of PTOA, zebrafish offer the advantage of live deep skeletal tissue imaging in response to changes in load or inhibition of miR signalling.
